# The *Viscum album* Gene Space database

**DOI:** 10.3389/fpls.2023.1193122

**Published:** 2023-06-26

**Authors:** Lucie Schröder, Oliver Rupp, Michael Senkler, Nils Rugen, Natalija Hohnjec, Alexander Goesmann, Helge Küster, Hans-Peter Braun

**Affiliations:** ^1^ Plant Proteomics, Institute of Plant Genetics, Leibniz Universität Hannover, Hannover, Germany; ^2^ Bioinformatics and Systems Biology, Justus-Liebig-Universität Gießen, Gießen, Germany; ^3^ Plant Genomics, Institute of Plant Genetics, Leibniz Universität Hannover, Hannover, Germany

**Keywords:** database development, PacBio sequencing, Illumina sequencing, Complexome profiling, mitochondria, oxidative phosphorylation (OXPHOS), complex I, supercomplex

## Abstract

The hemiparasitic flowering plant *Viscum album* (European mistletoe) is known for its very special life cycle, extraordinary biochemical properties, and extremely large genome. The size of its genome is estimated to be 30 times larger than the human genome and 600 times larger than the genome of the model plant *Arabidopsis thaliana*. To achieve insights into the Gene Space of the genome, which is defined as the space including and surrounding protein-coding regions, a transcriptome project based on PacBio sequencing has recently been conducted. A database resulting from this project contains sequences of 39,092 different open reading frames encoding 32,064 distinct proteins. Based on ‘Benchmarking Universal Single-Copy Orthologs’ (BUSCO) analysis, the completeness of the database was estimated to be in the range of 78%. To further develop this database, we performed a transcriptome project of *V. album* organs harvested in summer and winter based on Illumina sequencing. Data from both sequencing strategies were combined. The new *V. album* Gene Space database II (VaGs II) contains 90,039 sequences and has a completeness of 93% as revealed by BUSCO analysis. Sequences from other organisms, particularly fungi, which are known to colonize mistletoe leaves, have been removed. To evaluate the quality of the new database, proteome data of a mitochondrial fraction of *V. album* were re-analyzed. Compared to the original evaluation published five years ago, nearly 1000 additional proteins could be identified in the mitochondrial fraction, providing new insights into the Oxidative Phosphorylation System of *V. album*. The VaGs II database is available at https://viscumalbum.pflanzenproteomik.de/. Furthermore, all *V. album* sequences have been uploaded at the European Nucleotide Archive (ENA).

## Introduction

European mistletoe (*V. album*) is an obligate hemiparasitic flowering plant of the order Santalales. It grows on numerous trees in Europe. The host trees provide *V. album* with water, nutrients and, to a certain extent, with organic compounds. At the same time, *V. album* performs photosynthesis and produces organic compounds itself. In contrast to most angiosperms in Central Europe, it does not discard its leaves in winter and performs photosynthesis all year. The vitality of host trees may be impaired by mistletoe settlement.

*V. album* is known for a very special lifestyle (see Glatzel and Geils, 2009 for review): *V. album* does not germinate in soil but on branches of trees where it becomes connected with the xylem of its host. To ensure spreading, the fruits of *V. album* ripe in winter, when other food resources are scarce for birds. The fruits are very sticky to ensure a stable attachment on branches. The seeds lack a seed coat and consist of an embryo, which can germinate from the fruit without a dormancy phase. Haustoria, which are formed first during germination, are guided *via* negative phototropism to the surface of the branch of the host tree. After connection with the xylem of the vascular system, the haustoria take up water, minerals and organic compounds from the host plants. Afterwards, one pair of shoot segments per year per shoot apical meristem are formed by the *V. album* plant. The typical ball-like shape is achieved after several years by annual realignment of the new shoots, which grow in all directions. In contrast to most other plant species the leaves of *V. album* do not close their stomata during water shortage, which can increase water stress of the host plant. In August/September leaves of the previous year are discarded, without recycling chlorophyll, while the new leaves formed in spring stay attached, to perform photosynthesis in the winter.

Biochemically, *V. album* stands out with its rich content of phenolic acids, phenylpropanoids, flavonoids, triterpenes and phytosterols (Urech and Baumgartner, 2015; Jäger et al., 2021). Furthermore, *V. album* produces specific proteins, the viscotoxins and mistletoe lectins, which act as a biotic defense system. The stickiness of the fruits is provided by special kinds of hemicellulose compounds (Azuma Ji et al., 2000). The development of *V. album* is controlled by an extraordinary distribution of phytohormones. *V. album* extracts have immune stimulating and cytotoxic effects, which are used in medicine (Nazaruk and Orlikowski, 2016).

At the molecular level, *V. album* is less well characterized. The mitochondrial and chloroplast genomes have been sequenced (Petersen et al., 2015a; Petersen et al., 2015b; Skippington et al., 2015; Skippington et al., 2017) and found to lack some of the genes normally present in these organelles, especially those encoding subunits of the mitochondrial NADH dehydrogenase complex and the homologous chloroplastidic NDH complex. The complete absence of these complexes was shown by proteomic studies (Maclean et al., 2018; Senkler et al., 2018; Schröder et al., 2020; Schröder et al., 2022a).

The genome of *V. album* consists of 2n=20 chromosomes and is considered to be one of the larges genomes of flowering plants (Zonneveld, 2010; Novák et al., 2020). It consists of almost 100 billion base pairs. More than 50% of the genome sequence of *V. album* consists of highly repetitive DNA (Novák et al., 2020). The genome sequence of *V. album* has not been determined to date but the partial sequence of its Gene Space has recently been presented (Schröder et al., 2022b). The GC content of the gene sequences lies at about 50%, which is exceptionally high for angiosperms.

An initial approach to analyze the *V. album* Gene Space was based on Single Molecule Real-Time (SMRT) sequencing (PacBio sequencing) (Schröder et al., 2022b). A database resulting from this project contains 39,092 gene sequences, from which 32,064 protein sequences were derived. The results enabled the development of a first *V. album* Gene Space database. The completeness of this database was estimated to be in the range of 78%. To further develop this database, a *V. album* transcriptome project has been carried out using the Illumina sequencing approach. Analyses were performed for *V. album* samples harvested in winter and summer, respectively. By combining novel and existing sequencing data, we here present a new *V. album* database including 90,039 sequence entries. The quality of the new database is demonstrated by re-evaluation of published *V. album* proteome datasets.

## Materials and methods

### Isolation of mRNA fractions from *V. album*

Mistletoes (European mistletoe; *Viscum album*), grown on an apple tree (*Malus* sp.) on the campus of Leibniz University Hannover were used as starting material. Various organs (leaves, stems, flower buds) of male and female plants were harvested in summer and in winter, shock-frozen using liquid nitrogen, and stored at –80°C until use. mRNA isolation and quality evaluation were carried out as described previously (Schröder et al., 2022b).

### Sequence analysis

mRNA fractions were reverse transcribed into cDNA. The summer and winter fractions were analyzed separately using Illumina PE150 (paired-end read) sequencing. cDNA libraries containing 250~300 bp inserts were constructed and sequenced. Quantitative data as well as quality evaluation data of the libraries are given in **Table 1**.

**Table 1:** Results and quality evaluation of the Illumina sequencing approach of the winter and summer samples of *V. album*.

Sample^a^	Raw reads^b^	Raw data(G)^c^	Error (%)^d^	Q20 (%)^e^	Q30 (%)^f^	GC (%)^g^
winter	120,720,364	18,108,054,600	0.03	97.96	93.99	49.47
summer	121,840,742	18,276,111,300	0.02	98.11	94.34	49.37

a sample names.

b original sequencing reads counts.

c raw reads number multiplied by read lengths, saved in G unit.

d average sequencing error rate, calculated by Qphred=-10log10(e).

e percentages of bases whose correct base recognition rates are greater than 99% in total bases.

f percentages of bases whose correct base recognition rates are greater than 99.9% in total bases.

g percentages of G and C in total bases.

### Transcriptome assembly

The raw Illumina RNA-seq reads were first trimmed for low-quality and adaptor regions using Trimmomatic (Bolger et al., 2014) (version 0.36, ILLUMINACLIP : ADAPTER.fa:2:20:7 SLIDINGWINDOW:4:15 MINLEN:50). The trimmed reads were assembled using Trinity (Haas et al., 2013) (version 2.11.0) including *in silico* normalization with a target coverage of 50 x. The PacBio IsoSeq reads were included in the assembly using the “–long_reads” option of Trinity. The assembled transcripts were screened for coding sequences using TransDecoder (Haas and Papanicolaou, n.d.). The potential protein sequences were merged with the previously created protein sequences (Schröder et al., 2022b) and redundant sequences were filtered using CD-HIT (Li and Godzik, 2006) (version 4.7, -c 0.95 -aS 0.99). The functional annotation was computed using the best blast hit method on the UniProt/Swiss-Prot (Boutet et al., 2007) and UniProt/TrEMBL (The UniProt Consortium et al., 2023) databases. The TPM values were computed with Salmon (Patro et al., 2017) (version 1.9.0). The completeness of the filtered transcripts was estimated with BUSCO (Seppey et al., 2019) (version 5.0.0) using the viridiplantae database.

### The *V. album* database

The final *V. album* Gene Space II database was created by combining PacBio and Illumina sequencing. It includes 90,039 distinct proteins. The database is accessible at https://viscumalbum.pflanzenproteomik.de/. Furthermore, all sequences were submitted to NCBI.

### Re-evaluation of the *V. album* mitochondria complexome

The complexome profiling approach is introduced in Schröder et al., 2022c. Primary complexome profiling data were taken from Senkler et al., 2018. Re-evaluation of the mass-spectrometry (MS) data and annotation of proteins were carried out with MaxQuant (version 2.1.4.0) using the novel VaGs II database. The settings were the same as in Senkler et al., 2018. For heatmap generation, the abundance profiles (based on iBAQ values; Schwanhäusser et al., 2011; calculated by MaxQuant) of all identified proteins were used. These profiles were aligned according to similarity by hierarchical clustering using the NOVA software (version: 0.5.8; Giese et al., 2015). The Complexome Map of the re-evaluated *V. album* mitochondria fractions is available at the ComplexomeMap portal at https://complexomemap.de/75.

## Results and discussion

For Illumina analysis, transcripts from leaves, stems and buds of female and male *V. album* plants were isolated, combined and reverse-transcribed into cDNA. Organs were harvested in winter and summer and corresponding cDNA fractions were analyzed separately. Illumina sequence analysis revealed >120,000,000 reads for the summer and the winter fraction, respectively (**Table 1**).

### Transcriptome assembly

The datasets were processed as described in the Materials and Methods section and assembled using the Trinity software package version 2.11.0 (Haas et al., 2013). The PacBio IsoSeq reads (Schröder et al., 2022b) were included to the assembly using the “–long_reads” option of Trinity. The Trinity assembly produced 650,594 transcript contigs. TransDecoder predicted potential coding sequences on 144,517 contigs. The clustering with CD-HIT produced 104,405 unique sequences, which were used for further analyses.

### Functional annotation of transcripts and functional evaluation of the *V. album* transcript samples

Functional annotation of the 104,405 transcripts was computed using the best blast hit method on the UniProt/Swiss-Prot (Boutet et al., 2007) and UniProt/TrEMBL (The UniProt Consortium et al., 2023) databases. As a result, 51% of the sequences could be assigned a function. As expected, in most cases the greatest similarity is with sequences from the phylum *Viridiplantae* (green algae and the land plants). However, there are also numerous transcripts which show highest similarity to transcripts from other phyla, especially fungi. No functions could be assigned to many other sequences, which can likely be explained by the fact that no genome sequences of plants more closely related to *V. album* are yet available.

**Figure 1:**
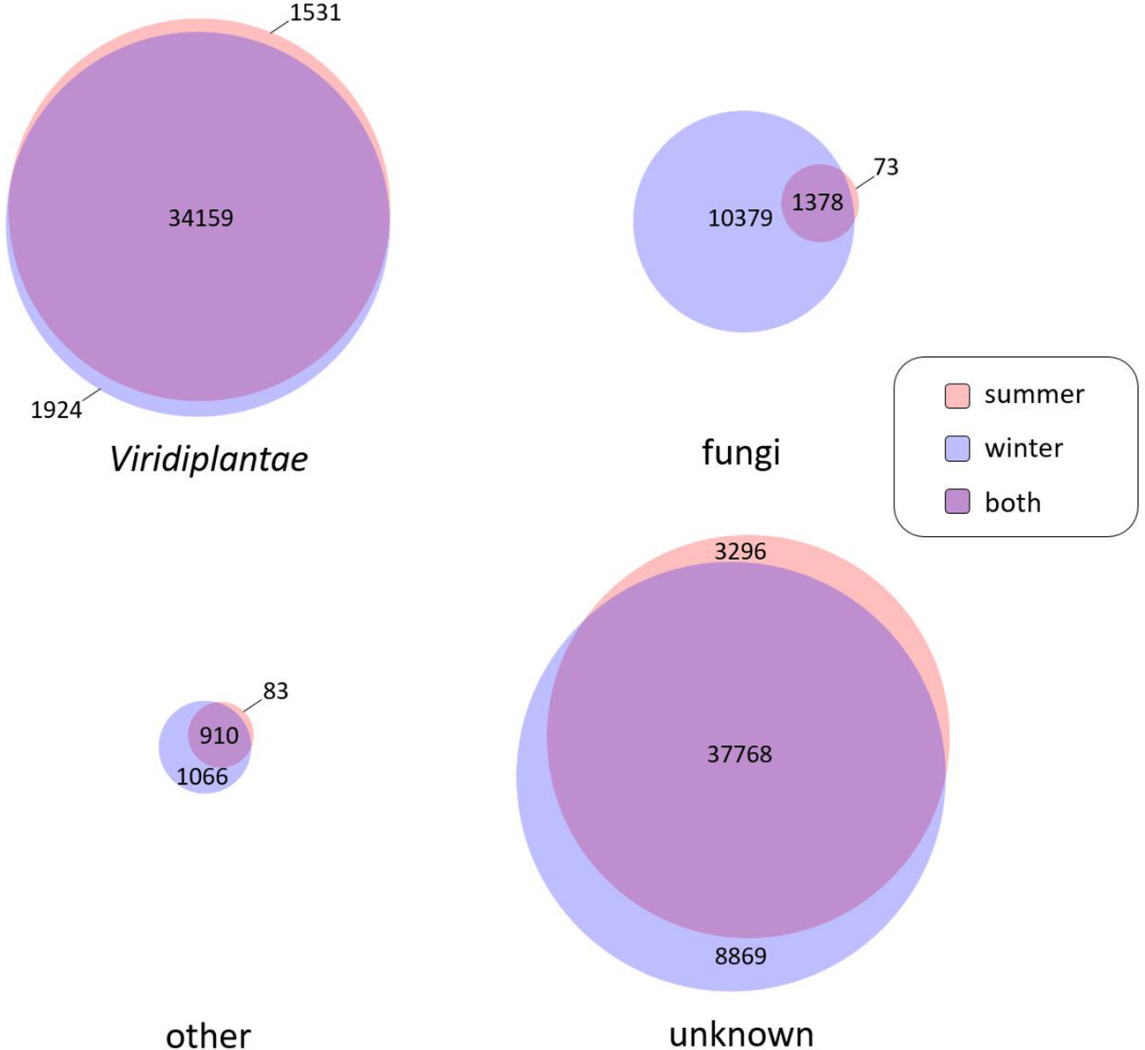
Number of transcripts according to assignment to phyla. Assignment of transcripts obtained by Illumina Sequencing was based on the best blast hit method on the UniProt/Swiss-Prot and UniProt/TrEMBL databases. Number of transcripts of summer and winter samples were displayed separately. Phyla considered are *Viridiplantae* (green algae and the land plants (embryophytes)) and fungi (as yeasts, molds and mushrooms). Transcripts resembling those of other phyla (e.g.: metazoan, bacteria etc.) were grouped under “other”. Transcripts not significantly resembling known sequences were labeled “unknown”. The Venn diagram was created by BioVenn (Hulsen et al., 2008).

*V. album* organs used for transcript isolation were from plants living in the field. *V. album* leaves are known to be colonized by several endophytic fungi (Peršoh et al., 2010; Kotan et al., 2013, Ariantari et al., 2019, reviewed in Krasylenko et al., 2020). To investigate the presence of fungal sequences in our database, we evaluated the functional annotation of Illumina transcript sequences according to Phyla for the winter and summer samples (**Figure 1**). 37.614 of the *V. album* transcripts assigned to *Viridiplantae*. 91% of them (34.159 transcripts) likewise were present in both, the winter and the summer sample. Furthermore, 11.830 transcripts of our *V. album* samples were assigned to the fungi Phylum (**Figure 1**). Almost all fungal sequences are from the winter sample, which is probably due to the increased age of the mistletoe leaves (new leaves appear in the spring and are kept in the winter). Only 11% of the fungal sequences occur likewise in the summer and winter samples. Few of the transcripts of our *V. album* samples were assigned to other Phyla than *Viridiplantae* or fungi (**Figure 1**). Transcripts that could not be functionally annotated were labeled “unknown” (**Figure 1**). The number of transcripts of this category is similar in the winter and summer samples; also, they show quite a high intersection in terms of their occurrence in the two seasons (76%), similar to the transcripts assigned to *Viridiplantae*. We conclude that these sequences should be predominantly *V. album* transcripts. The relatively high proportion of transcripts of unknown function likely is due to the lack of comparative sequences from related plant species, but also due to the fact that *V. album* has a special way of life, which requires numerous proteins that do not occur in other phyla.

**Figure 2:**
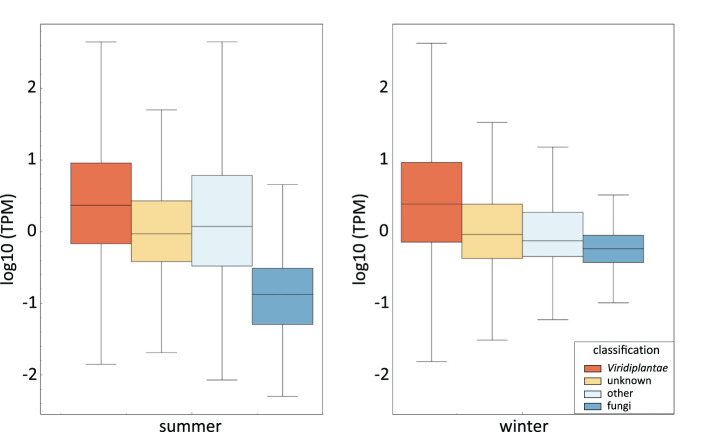
Transcript levels within the *V. album* summer and winter samples according to their assignment of phyla. Transcript levels are given in log10 Transcripts Per Million (TPM). The line within the box shows the median for each dataset. The upper end of the box is the “upper quartile”, which is the median of the upper half of the dataset. The lower quartile (lower end of the box) is the median of the lower half of the dataset. The end of the vertical lines indicate the lowest and highest values of the dataset. The diagram was created by instant clue (Nolte et al., 2018).

To further analyze the origin of sequences in our *V. album* samples, we quantified all transcripts identified by our Illumina sequencing approach according to their assignment to Phyla (**Figure 2**). Transcripts per million (TPM) values of transcripts in the summer and winter samples were examined separately. As expected, TMP values are highest for transcripts assigned to *Viridiplantae* and clearly lower in those assigned to the fungi Phylum. This is particularly visible in the sample harvested in summer. TMP values of unassigned transcripts more resemble the values obtained for transcripts assigned to *Viridiplantae*, which is especially evident in the summer sample. This reconfirms that the transcripts in this category are derived from *V. album* rather than from fungi colonizing *V. album*. The absolute summed up TPM values (summer and winter sample combined) for the four categories are 1,096,579 for *Viridiplantae* (73%), 385,858 for “unknown” (25%), 11,497 for fungi (0.8%) and 17,789 for “others” (1.1%). Thus, about 1% of the transcripts of our *V. album* fractions can be classified as fungal. This should realistically reflect the conditions in the “ecological niche” *V. album*.

### The *Viscum album* Gene Space database II

**Figure 3:**
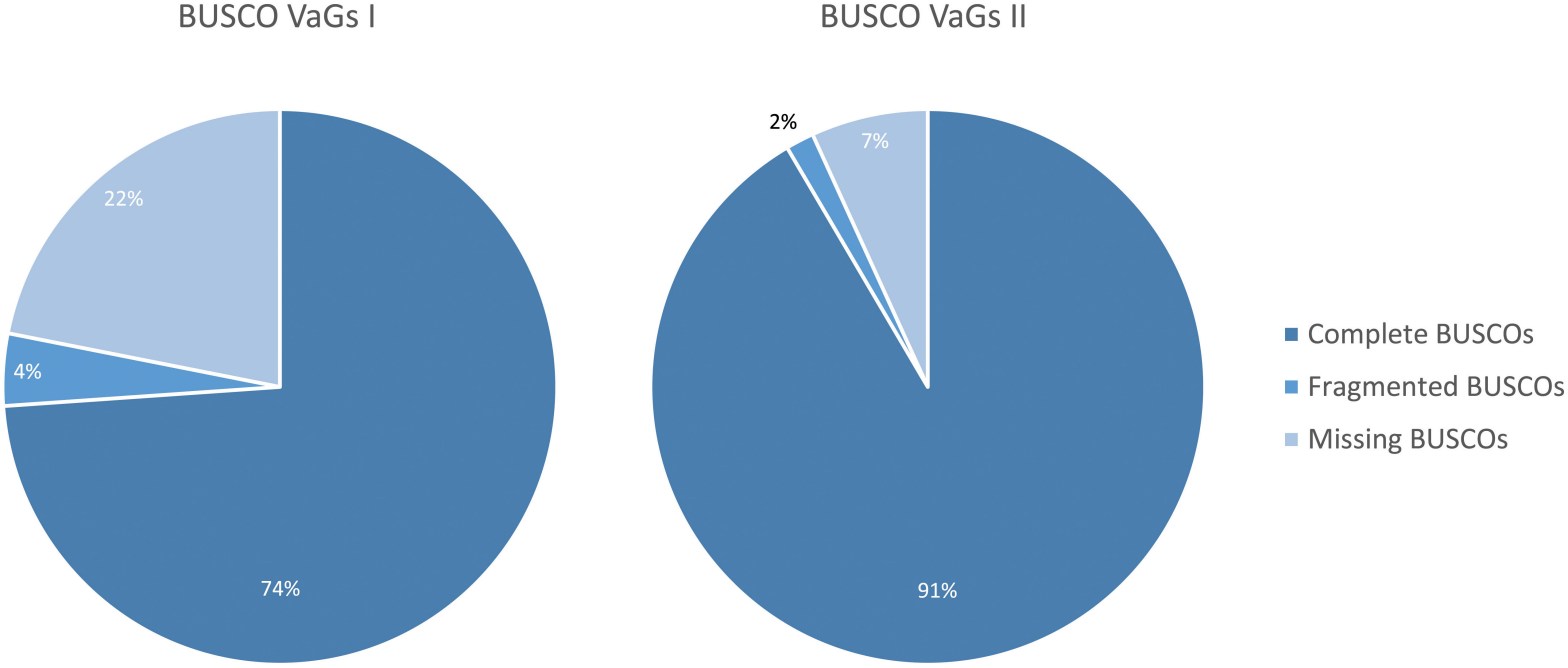
Comparison of the completeness of the VaGs I database (Schröder et al., 2022b) and the new VaGs II database as revealed by ‘Benchmarking Universal Single Copy Orthologs’ (BUSCO) analysis (Seppey et al., 2019).

The previous *V. album* Gene Space database, which was created based on Single Molecule Real-Time (SMRT) sequencing (PacBio sequencing), includes 39,092 entries encoding 32,064 distinct proteins (Schröder et al., 2022b). We now designate this database VaGs I. BUSCO analyses had revealed a completeness of about 78%. Through implementation of the new Illumina sequencing data and improved transcript annotation, we hereby present a novel *V. album* Gene Space database, VaGs II. We decided to remove transcripts that are unambiguously assigned to the Phyla Fungi, Animals, Bacteria and Viruses based on sequence comparisons. VaGs II includes all transcripts assigned to the categories “*Viridiplantae*” and “unknown”. The totel number of distinct transcripts is 90,039. The BUSCO score of VaGs II is 93% (91% complete, 2% fragmented; **Figure 3**).

**Figure 4:**
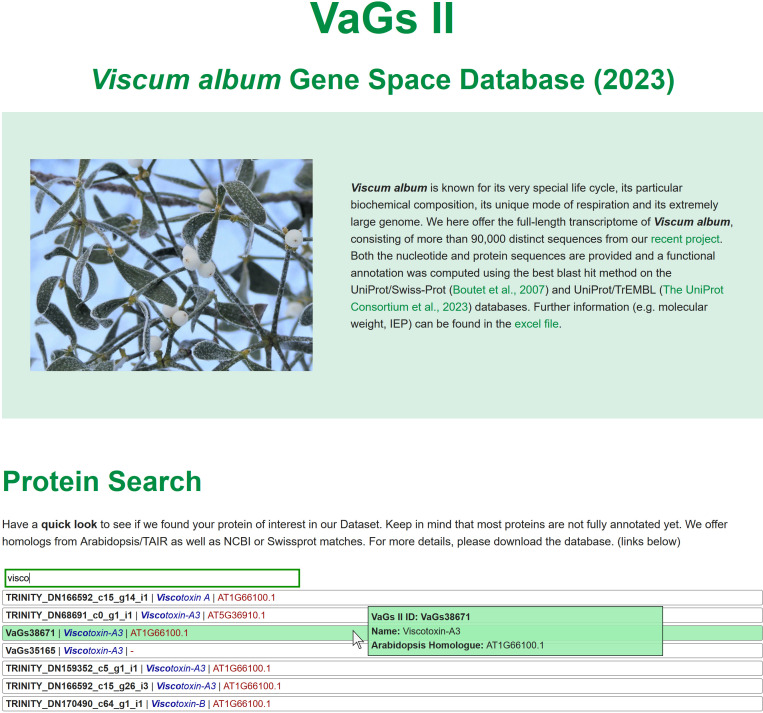
The *Viscum album* Gene space database at https://viscumalbum.pflanzenproteomik.de/.

A database has been developed that allows all sequences to be viewed (**Figure 4**). Sequences can be downloaded, proteins can be searched by ID, name, and sequence, and instructions for BLAST searches are given. Furthermore, all *V. album* transcript have been uploaded at the European Nucleotide Archive (ENA) (https://www.ebi.ac.uk/ena/browser/home).

### Re-evaluation of complexome profiling data for *V. album* mitochondria using VaGs II

**Figure 5:**
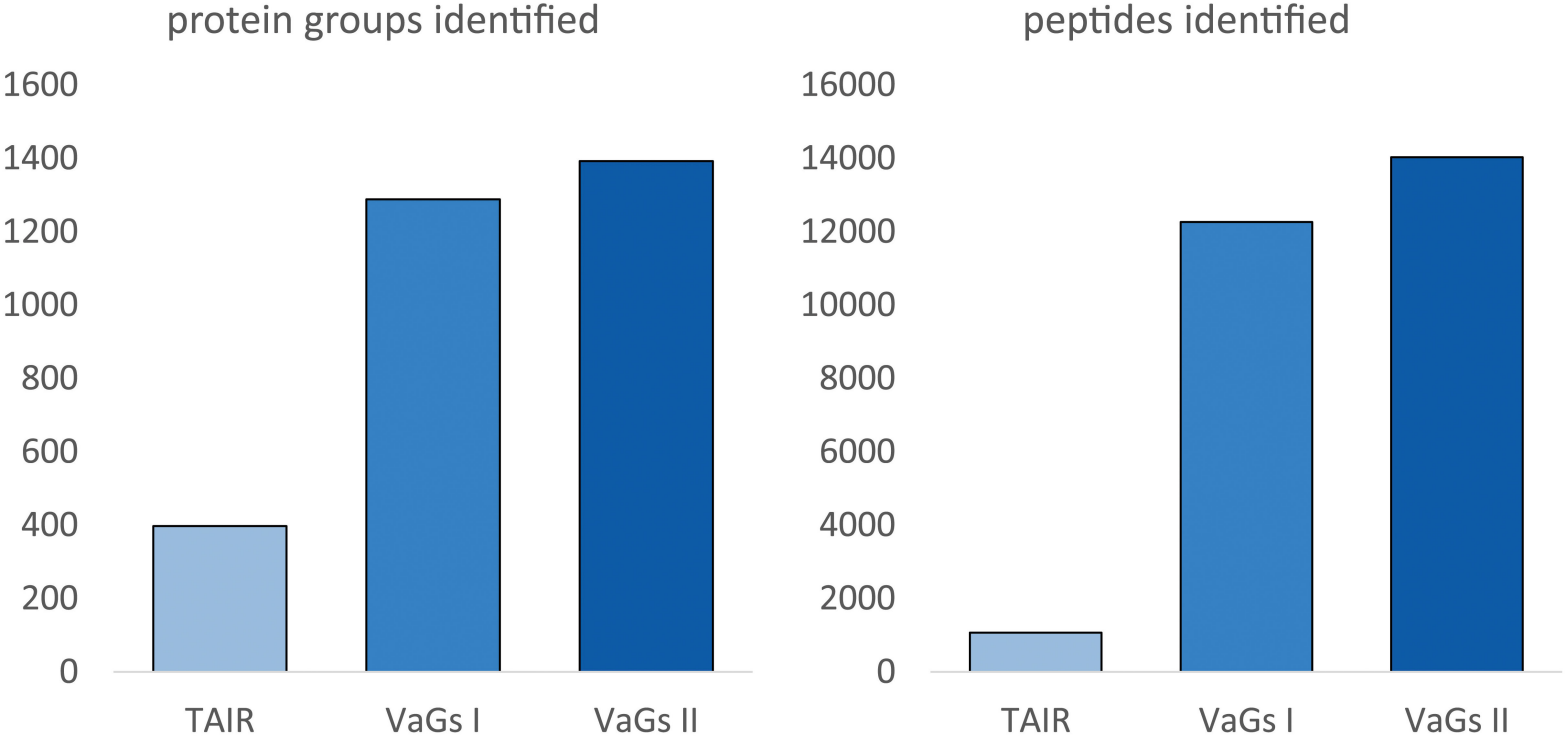
Number of identified proteins or peptides in a *V. album* mitochondrial complexome dataset upon data evaluation with TAIR, VaGs I and VaGs II. TAIR10: Arabidopsis protein database provided by The *Arabidopsis thaliana* Information resource (TAIR), Version 10 (https://www.arabidopsis.org/). The mitochondrial complexome dataset is derived from Senkler et al., 2018. The dataset was evaluated by TAIR10 (as in Senkler et al., 2018), VaGs I (Schröder et al., 2022b) and VaGs II (this study).

To test the quality of the new VaGs II databank, a published proteomic dataset on *V. album* mitochondria (Senkler et al., 2018) was re-evaluated. This is a complexome dataset which was created as follows: First, mitochondria were isolated from *V. album* and a mild detergent was used to dissolve the mitochondrial membranes. Protein complexes in the resulting solution were separated by Blue-native polyacrylamide gel electrophoresis. A lane of the native gel was then cut from top to bottom into 54 small gel slices, which were individually subjected to label-free quantitative shotgun mass spectrometry for protein identifications. Finally, abundance profiles along the blue-native gel lane were calculated for all identified proteins. The results were visualized as a heatmap and the profiles of individual proteins were aligned according to similarity. On the resulting heatmap, proteins belonging to the same protein complex form characteristic clusters. It thus is possible to systematically investigate the protein complexes of a biochemical fraction, the ‘complexome’. The original data of this experiment still had to be analyzed using a protein database for the model plant *Arabidopsis thaliana* (Senkler et al., 2018), since a *V. album* database was not available. This allowed the identification of 477 proteins in total. Re-evaluation of the same dataset using the VaGs II database now allowed the identification of 1392 proteins (**Figure 5**). The newly evaluated mitochondrial complexome of *V. album* is presented as a supplement of this publication (**Supplementary Table 1**) and is also accessible at the ComplexomeMap portal at https://complexomemap.de/75. It offers novel insights into the molecular biology of *V. album* mitochondria. Also, the number of peptides identified in the complexome fractions (gel slices) significantly increased based on VaGs II evaluation, resulting in a better coverage of proteins by peptides.

**Figure 6:**
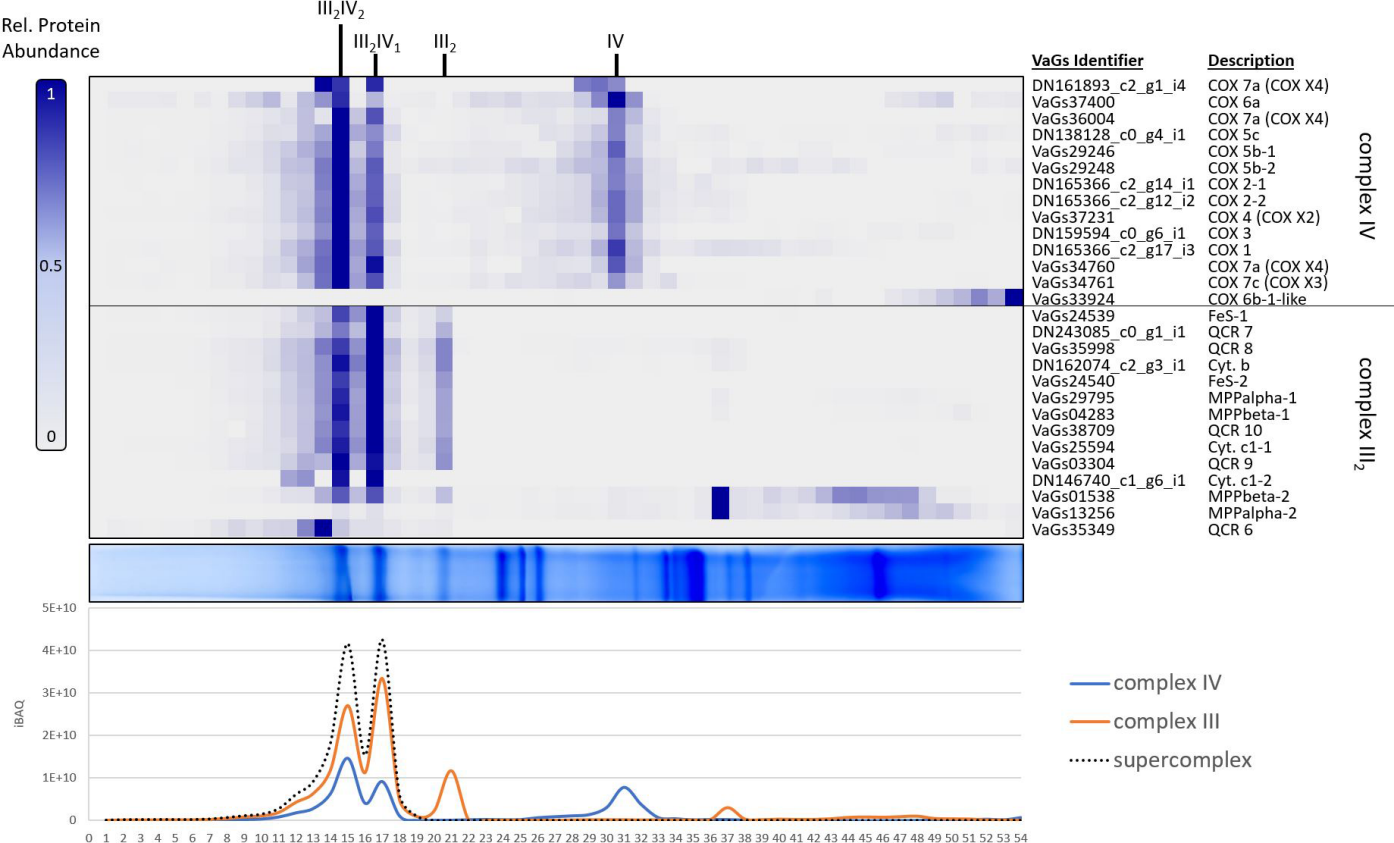
Identified subunits of complexes III and IV of the respiratory chain from *V. album* in a mitochondrial complexome dataset (Senkler et al., 2018) upon analysis using the VaGs II database. Mitochondrial proteins were separated by Blue native (BN) PAGE and stained by Coomassie-blue (horizontal gel lane in the center of the Figure). The gel lane was dissected into 54 slices, which all were subjected to label-free quantitative shotgun mass spectrometry. Abundance profiles of individual proteins along the gel lane are visualized as a heatmap (figure part above the gel lane; the columns correspond to the 54 gel slices; the rows to the abundance profiles of individual proteins; normalized (max) abundance profiles are given; see scale to the left of the heatmap) and aligned according to similarity (using the NOVA software package version 0.5.8). Only profiles of the subunits of respiratory complexes III and IV are shown (for complete dataset see **Supplementary Data S1** and ComplexomeMap at https://complexomemap.de/75). Protein identities are given to the right of the heatmap. Summed-up abundance profiles (given as ‘intensity based absolute quantification’ (iBAQ) values) of the subunits of the two protein complexes as well as assemblies of the complexes (supercomplexes III_2_IV_1_ and III_2_IV_2_) are given in the diagram (figure part below the gel lane; y-axis: iBAQ score; x-axis: gel slices 1-54). Results of the original evaluation (Senkler et al., 2018) are shown in **Supplementary Figure S1**).

To further analyze the mitochondrial complexome from *V. album*, as revealed by VaGs II evaluation, we took a closer look at the protein clusters corresponding to respiratory chain complexes III and IV (**Figure 6**). Complex III is an ubiquinol:cytochrome-c oxidoreductase and complex IV a cytochrome-c:O_2_ oxidoreductase. These two protein complexes catalyze the last two steps of the mitochondrial respiratory chain and were found to form two exceptionally stable supercomplexes in *V. album* mitochondria (Senkler et al., 2018). According to current knowledge (Maldonado et al., 2021), complexes III and IV in plants each consist of 10 different subunits (the 10 subunits of complex III, however, all are present in duplicate, as complex III occurs as a functional dimer). In *V. album*, this dimer (III_2_) associates with one or two copies of complex IV; the corresponding supercomplexes are designated III_2_IV_1_ and III_2_IV_2_. Of the total 20 different proteins present in these supercomplexes, all 20 proteins were found in our complexome dataset upon evaluation using VaGs II (**Figure 6**; compared to only 8 proteins based on the original evaluation, Senkler et al., 2018; see **Supplementary Figure S1**). 18 of the identified subunits form part of the two supercomplexes, whereas two (QCR6 of complex III_2_ and Cox6b from complex IV) are partly detached and migrate in the low-molecular-mass region of the blue native gel, probably because they became detached during membrane solubilization. For some of the proteins, different isoforms are present in *V. album* (e.g. the Cox5b and the α- and β-MPP subunits, **Supplementary Figures S2**, **S3**). The summed-up abundance profiles of the subunits of complex III and of complex IV reveal that the amounts of the two supercomplexes are similar; furthermore, the stoichiometry of the two complexes within the supercomplexes is correctly revealed (**Figure 6**, bottom).

## Concluding remarks

We present here the *Viscum album* Gene Space database II, VaGs II. Based on our quantitative and qualitative evaluations, we assume that it covers well above 90% of the protein-coding genes of *V. album*. The database has been cleaned with respect to sequences from other organisms, particularly fungi that are known to colonize *V. album* leaves. The database contains 90,039 transcript sequences. In particular, the functional annotation of the sequences was improved. Moreover, winter and summer samples of *V. album* were studied separately based on Illumina sequencing. Further analysis of these data should provide insights into adaptations of *V. album* to different seasons. VaGs II should provide an important data background useful for further studying the molecular biology of this extraordinary plant.

## Data availability statement

The new VaGs II database is available at https://viscumalbum.pflanzenproteomik.de/. In addition, fasta files of thenucleotide or protein sequences can be downloaded. All Illumina and PacBio sequencing datasets and their assembly dataset have been uploaded to the European Nucleotide Archive (ENA) and can be found under the study identifier PRJEB60149. Within this study, the PacBio dataset can be found under the identifiers ERR10970196 and ERR10970197, the Illumina summer dataset under ERR10968077 and the Illumina winter dataset under ERR10968073. The original mass spectrometry proteomics data (Senkler et al., 2018) have been deposited to the ProteomeXchange Consortium via the PRoteomics IDEntifications database (PRIDE, Perez-Riverol et al., 2022) partner repository with the dataset identifier PXD008974. The re-evaluated dataset based on an evaluation using the new VaGs II database has been deposited additionally to the ProteomeXchange Consortium via PRIDE and is accessible under PXD041061.

## Author contributions

HPB, HK, and LS initiated and supervised the project. NH isolated mRNA from *V. album* organs. OR, AG, and LS carried out database development and data annotation. NR and LS re-evaluated complexome-profiling data using the new database. MS developed the web portal for the *V. album* Gene Space database. LS and HPB performed data evaluation and interpretation. LS and HPB wrote the manuscript. All authors contributed to the article and approved the submitted version.

## Supplementary material

Supplementary Figure 1 Identified subunits of complexes III and IV of the respiratory chain from V. album in a mitochondrial complexome dataset (Senkler et al., 2018) upon analysis using the Arabidopsis TAIR10 database (https://www.arabidopsis.org/). The same dataset was re-analyzed using the VaGs database (**Figure 6**).

Supplementary Figure 2 Sequence alignment of *V. album* COX5b-1 and COX5b-2. The COX 5b subunit of the cytochrome c oxidase complex (Complex IV) is present in two isoforms, COX5b-1 and COX5b-2. The proteins are encoded by transcripts VaGs29248 and VaGs29246. Conserved amino acids positions are highlighted in blue. Both proteins form part of the III_2_IV and III_2_IV_2_ supercomplexes in the mitochondrial complexome dataset (**Figure 4** and **Supplementary Data S1**).

Supplementary Figure 3 Identified pairs of isoforms of the two subunits of the mitochondrial processing peptidase (MPP) from *V. album* in a mitochondrial complexome dataset (Senkler et al., 2018) upon re-evaluation using the VaGs II database. Both, the α- and the β-MPP subunits form part of the cytochrome c reductase complex (complex III) in plants (Braun, 2020). The β-subunit is encoded by transcripts VaGs01538 and VaGs04283; the α-subunit by transcripts VaGs13256 and VaGs29795. The heatmap indicates the abundance profiles of the four proteins along a blue-native gel lane used for the complexome profiling analysis. The positions of complexes III and IV and their supercomplexes are indicated above the heatmap, the molecular masses of standard proteins below the heatmap. One isoform of the α- and the β-subunit of MPP forms part of the III_2_IV and III_2_IV_2_ supercomplexes, respectively. The other two isoforms seem to (partially) form a separate protein complex in the 110-160 kDa range, which might represent a soluble form of the heterodimeric MPP enzyme (detached from complex III). A soluble MPP enzyme has been described for mitochondrial matrix of animals and yeast (Braun and Schmitz, 1995), but possibly also occurs in *V. album*.

Supplementary Data Sheet 1 Heatmap of abundance profiles of the proteins included in the mitochondrial complexome dataset of *V. album* re-evaluated using the VaGs II database.
