# Supplementary material for: The Viscum album Gene Space database
Source: Front Plant Sci. 2023 Jun 26;14:1193122. doi: 10.3389/fpls.2023.1193122 (PMC10359728; doi:10.3389/fpls.2023.1193122)
Supplement: Supplementary Figure 1 — Identified subunits of complexes III and IV of the respiratory chain from V. album in a mitochondrial complexome dataset (Senkler et al., 2018) upon analysis using the Arabidopsis TAIR10 database (https://www.arabidopsis.org/). The same dataset was re-analyzed using the VaGs database (Figure 6). [file Image_1.pdf]

Supplemental Material, Schröder et al. 2023

Supp. Fig. 1

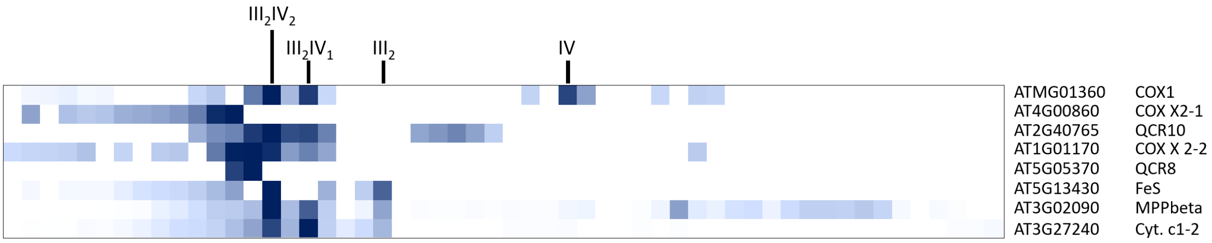

Supp. Fig. S1: Identified subunits of complexes III and IV of the respiratory chain from *V. album* in a mitochondrial complexome dataset (Senkler et al. 2018) upon analysis using the Arabidopsis TAIR10 database (https://www.arabidopsis.org/). The same dataset was re-analyzed using the VaGs database (Fig. 6)

Supp. Figure 2

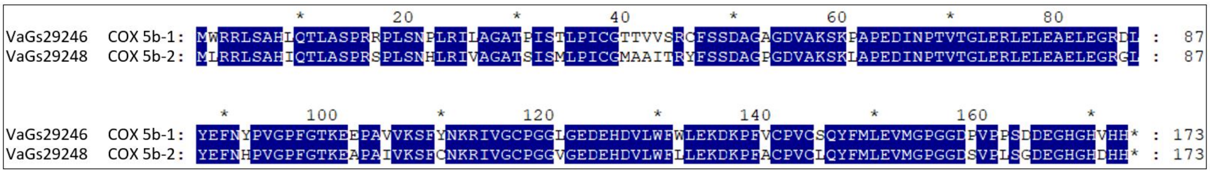

Supp. Figure S2: Sequence alignment of *V. album* COX5b-1 and COX5b-2. The COX 5b subunit of the cytochrome c oxidase complex (Complex IV) is present in two isoforms, COX5b-1 and COX5b-2. The proteins are encoded by transcripts VaGs29248 and VaGs29246. Conserved amino acids positions are highlighted in blue. Both proteins form part of the III_2IV and III_2IV_2 supercomplexes in the mitochondrial complexome dataset (Fig. 6 and Supp. data S1).

Supp. Figure S3

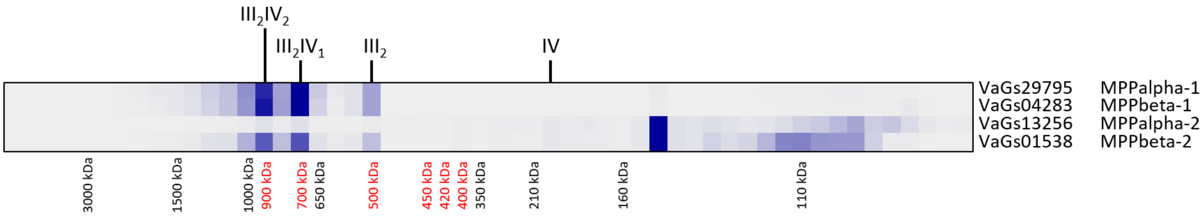

Supp. Figure S3: Identified pairs of isoforms of the two subunits of the mitochondrial processing peptidase (MPP) from *V. album* in a mitochondrial complexome dataset (Senkler et al. 2018) upon re-evaluation using the VaGs II database. Both, the α- and the β-MPP subunits form part of the cytochrome c reductase complex (complex III) in plants (Braun 2020). The β-subunit is encoded by transcripts VaGs01538 and VaGs04283; the α-subunit by transcripts VaGs13256 and VaGs29795. The heatmap indicates the abundance profiles of the four proteins along a blue-native gel lane used for the complexome profiling analysis. The positions of complexes III and IV and their supercomplexes are indicated above the heatmap, the molecular masses of standard proteins below the heatmap. One isoform of the α- and the β-subunit of MPP forms part of the III_2IV and III_2IV_2 supercomplexes, respectively. The other two isoforms seem to (partially) form a separate protein complex in the 110-160 kDa range, which might represent a soluble form of the heterodimeric MPP enzyme (detached from complex III). A soluble MPP enzyme has been described for mitochondrial matrix of animals and yeast (Braun and Schmitz 1995), but possibly also occurs in *V. album*.
